# Heme Modulates Bladder Contractility Through the HO–CO–sGC–cGMP Pathway: Insights into Sickle Cell Disease-Associated Bladder Dysfunction

**DOI:** 10.3390/antiox14121398

**Published:** 2025-11-25

**Authors:** Dalila Andrade Pereira, Fernando Ferreira Costa, Fábio Henrique Silva

**Affiliations:** 1 Laboratory of Pharmacology, São Francisco University Medical School, Bragança Paulista 12916-900, SP, Brazil; 2 Hematology and Hemotherapy Center, University of Campinas, Campinas 13083-970, SP, Brazil

**Keywords:** carbon monoxide, cyclic GMP, smooth muscle, hemolysis, heme oxygenase

## Abstract

Intravascular hemolysis, a hallmark of sickle cell disease (SCD), leads to elevated plasma heme levels. Although heme is essential for physiological processes, its excess can be deleterious. Heme oxygenase (HO) degrades heme into carbon monoxide (CO), which activates the soluble guanylate cyclase (sGC)–cyclic guanosine monophosphate (cGMP) signaling cascade and can modulate smooth muscle tone. However, the direct effects of heme on bladder function remain unknown. This study investigated whether heme regulates detrusor smooth muscle contractility through the HO–CO–sGC–cGMP pathway. Detrusor strips from C57BL/6 mice were mounted on a myograph for functional analysis. Heme induced a significant, concentration-dependent relaxation of detrusor smooth muscle compared with vehicle-treated tissues. To elucidate the underlying mechanism, tissues were pre-incubated with the sGC inhibitor ODQ (10 µM) or the HO inhibitor 1J (100 µM) before heme exposure. Both inhibitors markedly attenuated heme-induced relaxation, reducing the maximal relaxation response. Moreover, pre-incubation with heme (100 µM) significantly decreased the maximal contractile responses (Emax) to carbachol, KCl, and electrical field stimulation (EFS), effects that were abolished by ODQ or 1J. In parallel, biochemical assays showed that heme markedly increased cGMP levels in detrusor tissue, an effect prevented by both inhibitors, confirming the role of the HO–CO–sGC–cGMP signaling cascade in this response. These findings demonstrate that heme modulates bladder contractility by activating the HO–CO–sGC–cGMP pathway, promoting detrusor relaxation. This mechanism suggests that excessive circulating heme, as occurs in hemolytic disorders such as SCD, may contribute to detrusor hypocontractility and voiding dysfunctions, identifying this pathway as a potential therapeutic target.

## 1. Introduction

Sickle cell disease (SCD) is a hereditary hemoglobinopathy characterized by the presence of abnormal hemoglobin S (HbS), which polymerizes under low oxygen conditions, resulting in rigid and sickle-shaped erythrocytes prone to hemolysis [1,2]. The resulting chronic hemolytic state, vascular occlusion, and ischemia–reperfusion injury contribute to progressive multi-organ damage and dysfunction [1]. Among the affected systems, the genitourinary tract has gained attention due to the frequent occurrence of lower urinary tract symptoms (LUTS), such as urinary incontinence, nocturia, and overactive bladder (OAB) [3,4,5]. OAB is clinically characterized by urgency or increased daytime urinary frequency, generally with small voided volumes, often associated with urgency incontinence. These symptoms can occur even in young individuals and are often underestimated in clinical practice. Epidemiological studies estimate that approximately 38% of SCD patients exhibit symptoms consistent with OAB, which may substantially impair sleep quality, social functioning, and overall quality of life [3,4,5].

In SCD, intravascular hemolysis releases substantial amounts of hemoglobin and free heme into plasma, overwhelming endogenous protective mechanisms such as haptoglobin and hemopexin, and consequently causing heme accumulation, as shown in studies in humans and mice [6,7,8,9,10,11]. Once released into circulation, free heme becomes loosely bound to plasma proteins and can easily diffuse into tissues, where it interacts with cell membranes and intracellular targets [8,12]. Although heme is indispensable for oxygen transport and cellular respiration, its excess exerts cytotoxic effects by promoting oxidative stress, lipid peroxidation, and cellular injury. Under such conditions, free heme acts as a pro-oxidant molecule capable of promoting endothelial dysfunction and inflammation in several tissues [12,13,14]. Collectively, these effects contribute to the disruption of vascular homeostasis and smooth muscle regulation, suggesting that heme overload may represent a key pathophysiological event in organ dysfunction associated with hemolytic disorders such as SCD.

To prevent heme-mediated toxicity, excess heme is enzymatically degraded by heme oxygenases (HO-1, inducible form; HO-2, constitutive form), yielding carbon monoxide (CO), biliverdin, and ferrous iron (Fe^2+^) [15]. This enzymatic system represents a crucial antioxidant and cytoprotective defense mechanism that limits oxidative injury and maintains tissue homeostasis under hemolytic stress. Among these metabolites, CO has gained attention as a biologically active gasotransmitter with homeostatic and cytoprotective properties, acting mainly through activation of soluble guanylate cyclase (sGC), thereby increasing cyclic guanosine monophosphate (cGMP) levels, a key second messenger that regulates smooth muscle tone [16,17,18,19,20,21,22,23,24,25,26]. Activation of this CO-sGC-cGMP signaling pathway mediates smooth muscle relaxation in several tissues, including vascular beds, urethral, and penile cavernous smooth muscle, potentially contributing to conditions such as priapism associated with SCD [27,28,29,30,31].

Despite clinical evidence indicating an association between SCD and lower urinary tract dysfunction, the underlying mechanisms remain poorly understood, and effective treatments are lacking [4,5,32,33,34,35,36,37]. It is plausible that repeated episodes of hemolysis, ischemia, and oxidative stress disrupt the delicate balance between contractile and relaxant mechanisms in the bladder wall, ultimately impairing detrusor performance. In this context, heme accumulation may represent a critical upstream event linking intravascular hemolysis to smooth muscle dysfunction. However, the specific role of excess heme and the potential involvement of the HO–CO–sGC–cGMP signaling pathway in modulating bladder contractility during SCD have not yet been elucidated. Clarifying these molecular interactions may provide new insights into how hemolysis-derived metabolites influence urinary physiology and could represent an important step toward developing targeted therapeutic strategies for bladder dysfunction associated with SCD.

Therefore, we hypothesized that elevated plasma heme levels contribute to detrusor muscle dysfunction observed in SCD via activation of the HO-CO-sGC-cGMP signaling pathway. To test this hypothesis, the present study investigated the direct effects of heme on the contractile and relaxant mechanisms of detrusor smooth muscle from healthy C57BL/6 mice, using a combination of pharmacological, functional, and biochemical approaches. This experimental design allowed us to isolate the specific actions of heme on smooth muscle regulation, independent of systemic alterations typically observed in sickle cell disease. Specifically, we evaluated whether heme exposure modifies contractile responses induced by carbachol, KCl, and electrical field stimulation (EFS), and whether these effects depend on the enzymatic activity of HO or sGC. In parallel, we quantified cGMP levels in detrusor tissue to confirm activation of the downstream signaling cascade. By integrating these functional and molecular analyses, the study sought to determine whether heme directly modulates bladder smooth muscle through the HO–CO–sGC–cGMP pathway. These findings are intended to provide mechanistic insight into how elevated circulating heme, such as that occurring during intravascular hemolysis in SCD, could contribute to detrusor hypocontractility and voiding dysfunction in vivo.

## 2. Materials and Methods

### 2.1. Animals

Male C57BL/6 mice (3–6 months old) were obtained from the Multidisciplinary Center for Biological Investigation (CEMIB, UNICAMP) and maintained at the Animal Facility of São Francisco University (USF) under controlled conditions (24 °C, 12 h light/dark cycle), with food and water available ad libitum. All experimental procedures were conducted in accordance with the ethical principles for animal experimentation and were approved by the Animal Use Ethics Committee of the University of São Francisco (CEUA-USF, protocol nº 008.06.2021, approved on 11 November 2021).

### 2.2. Preparation of Detrusor Muscle

Bladders were collected from mice anesthetized with an intraperitoneal injection of ketamine (100 mg/kg) and xylazine (10 mg/kg). All efforts were made to minimize animal suffering. After tissue collection, animals were euthanized by an overdose of isoflurane (12%). The bladder was rapidly excised and placed in Krebs–Henseleit solution composed of (mM): NaCl (130), NaHCO_3_ (14.9), dextrose (5.5), KCl (4.7), KH_2_PO_4_ (1.18), MgSO_4_·7H_2_O (1.17), and CaCl_2_·2H_2_O (1.6). The detrusor muscle was isolated by removing the bladder base and cutting longitudinal strips from the bladder body. The tissues were mounted in organ bath chambers (7 mL) containing Krebs–Henseleit solution, continuously aerated with carbogen (95% O_2_ and 5% CO_2_), and maintained at 37 °C, pH 7.4. Detrusor strips were suspended between a fixed support and an isometric force transducer (AD Instruments, Dunedin, New Zealand). Tissues were equilibrated under a resting tension of 5 mN for 60 min, and the solution was changed every 15 min. Contractile responses were recorded using PowerLab 4/35 data acquisition (AD Instruments, software version 7.0).

### 2.3. Concentration–Response Curves

Contraction responses were evaluated by cumulative addition of the muscarinic agonist carbachol (10 nM–100 µM) or KCl (1–300 mM) in detrusor strips pre-incubated for 30 min with heme (100 µM) or vehicle (0.1% dimethyl sulfoxide [DMSO]). DMSO is a commonly used solvent in pharmacological and toxicological research and is generally considered non-toxic at concentrations below 0.1% (*v*/*v*). It is acknowledged that the effects of DMSO are practically negligible [38].

To analyze the relaxation response, concentration–response curves to heme (10 µM–300 µM) were obtained in detrusor strips pre-incubated with DMSO. Before the addition of heme, tissues were precontracted with carbachol (300 nM) to reach a stable plateau of tension, since evaluation of relaxation ex vivo requires a precontracted state as a reference. Relaxation responses were also evaluated in tissues pre-incubated with 1J (HO inhibitor; 10 µM) or ODQ (sGC inhibitor; 10 µM) for 30 min before exposure to heme (100 µM) or vehicle (DMSO, 0.1%). The relaxation response was calculated as the percentage decrease in tension relative to the maximal contraction induced by carbachol (300 nM), which was taken as 100%. Contractile responses were expressed in milliNewtons (mN) per milligram of tissue (mN/mg).

Nonlinear regression analysis to determine the potency (pEC_50_) was carried out using GraphPad Prism 7 (GraphPad Software, San Diego, CA, USA) with the constraint that Φ = 0. All concentration–response data were evaluated for a fit to a logistics function in the form: E = Emax/([1 + (10^c^/10^x^)^n^] + Φ, where E is the maximum response produced by agonists; c is the logarithm of the EC_50_, the concentration of drug that produces a half-maximal response; x is the logarithm of the concentration of the drug; the exponential term, n, is a curve-fitting parameter that defines the slope of the concentration–response line, and Φ is the response observed in the absence of added drug. Potency (pEC_50_) and maximal effect (E_max_) values.

### 2.4. EFS-Induced Neurogenic Contraction

For the investigation of neurogenic contractions, two parallel platinum electrodes were positioned on either side of the detrusor strips, ensuring uniform field stimulation throughout the tissue. EFS was applied using square-wave pulses (20 V, 1 millisecond pulse duration, 0.2 millisecond pulse interval, 10-s train duration) at frequencies of 1, 2, 4, 8, 16, and 32 Hz, with a 2 min interval between stimuli to allow complete recovery between contractions. This stimulation protocol reliably evokes frequency-dependent neurogenic contractions, allowing assessment of nerve-mediated contractile responses under controlled conditions.

Contractile responses induced by EFS were recorded in tissues pre-incubated with heme (100 µM) or vehicle (DMSO) for 30 min. In additional experiments, the role of the HO–CO–sGC–cGMP pathway was evaluated by pre-incubating tissues with 1J (HO inhibitor; 10 µM) or ODQ (sGC inhibitor; 10 µM) for 30 min before heme or vehicle exposure. Contractile activity was normalized to tissue weight and expressed as mN/mg.

### 2.5. Determination of cGMP Levels in Mouse Detrusor Homogenates

Detrusor tissues were incubated at 37 °C in Krebs solution aerated with carbogen, followed by stimulation with heme (100 µM, 30 min) or vehicle (DMSO, 0.1%). Some tissues were pre-incubated (30 min) with inhibitors ODQ (100 µM) or 1J (100 µM) before heme treatment. Sodium nitroprusside (SNP; 100 µM, 15 min) served as a positive control.

Tissues were immediately snap-frozen in liquid nitrogen, pulverized, and homogenized in 5% trichloroacetic acid (TCA). Samples were centrifuged (2000× *g*, 10 min, 4 °C), and TCA was extracted with water-saturated ether (3x). cGMP quantification was performed using the cGMP ELISA kit (Cayman Chemical, Ann Arbor, MI, USA), following the manufacturer’s instructions. Assays were conducted in duplicate.

### 2.6. Drugs and Chemicals

Heme, DMSO, carbachol, KCl, and ODQ were obtained from Sigma-Aldrich (St. Louis, MO, USA). Additional ODQ was acquired from Tocris Bioscience (Bristol, UK). Compound 1J was kindly provided by Prof. Valeria Pittalà (University of Catania, Sicily, Italy). All reagents were analytical grade, and solutions were freshly prepared immediately before experiments. Heme was initially dissolved in DMSO and subsequently diluted in Krebs–Henseleit buffer to obtain a final concentration of 100 μM heme and 0.1% (*v*/*v*) DMSO.

### 2.7. Statistical Analysis

The results are presented as mean ± standard error of the mean (SEM) from the indicated “n” experiments in each case. Unpaired Student’s *t*-tests were used for comparisons between two groups. For comparisons between three groups, one-way analysis of variance (ANOVA) was employed, followed by Tukey’s test. Values of *p* < 0.05 were considered significant.

## 3. Results

### 3.1. Heme Induces Detrusor Smooth Muscle Relaxation via the HO–CO–sGC–cGMP Pathway

**Figure 1:**
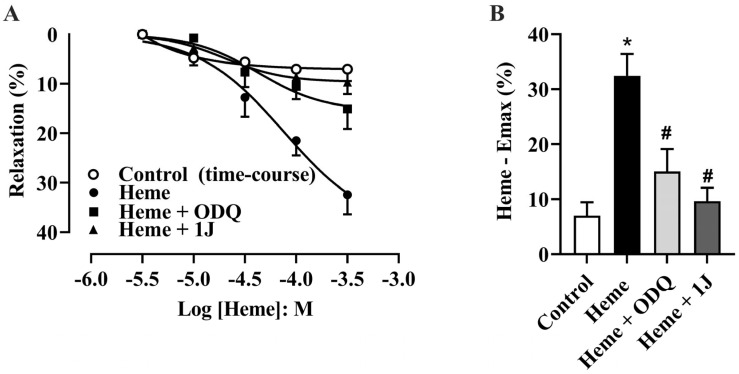
(**A**) Cumulative concentration–response curves for heme (10–300 µM) and the time-course control (vehicle, DMSO) in the absence or presence of the HO inhibitor 1J (100 µM) or the sGC inhibitor ODQ (10 µM). (**B**) Maximal relaxation values (Heme—Emax %), representing the percentage decrease in tension induced by heme relative to the maximal contraction produced by carbachol (300 nM), which was taken as 100%. The vehicle control accounts for both solvent effects and spontaneous relaxation over time. Data are expressed as the mean ± SEM from five independent experiments. Statistical analyses were performed using one-way analysis of variance (ANOVA) followed by Tukey’s post hoc test. * *p* < 0.05 vs. vehicle; # *p* < 0.05 vs. heme.

Detrusor smooth muscle relaxation was evaluated through cumulative concentration–response curves to heme (10–300 µM) in tissues pre-incubated with vehicle (DMSO, 0.1%). Heme produced a significant, concentration-dependent relaxation of detrusor strips compared with vehicle-treated tissues (*p* < 0.05; Figure 1A).

To identify the mechanism underlying heme-induced relaxation, detrusor tissues were pre-incubated with ODQ (10 µM, a sGC inhibitor) or 1J (100 µM, a non-selective HO inhibitor) for 30 min before the addition of heme. The presence of either ODQ or 1J significantly attenuated the heme-induced relaxation response, reducing the maximum effect (Emax, *p* < 0.05; Figure 1A,B). These results indicate that heme-induced relaxation of detrusor smooth muscle depends on activation of the HO–CO–sGC–cGMP pathway.

### 3.2. Heme Reduces Carbachol-Induced Detrusor Contraction via the HO–sGC Pathway

**Figure 2:**
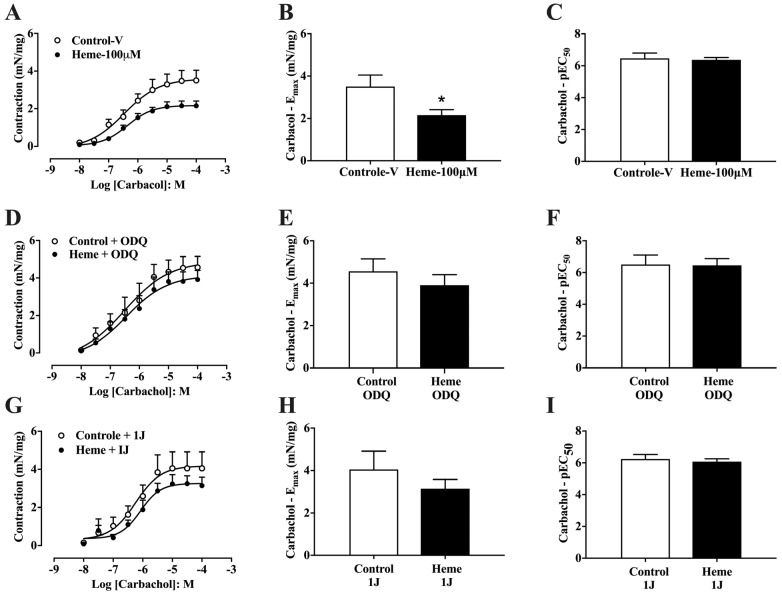
Concentration–response curves to carbachol in mouse detrusor smooth muscle: (**A**) Curves obtained in tissues pre-incubated with vehicle (Control–v) or heme (100 µM). (**D**) Curves obtained in the presence of the sGC inhibitor ODQ (10 µM) with or without heme (100 µM). (**G**) Curves obtained in the presence of the HO inhibitor 1J (100 µM) with or without heme (100 µM). (**B**,**E**,**H**) Maximal contractile responses (Eₘₐₓ, expressed as mN/mg of tissue) derived from the concentration–response curves shown in (**A**), (**D**), and (**G**), respectively. (**C**,**F**,**I**) Potency values (*p*EC_50_), calculated as the negative logarithm of the molar concentration of carbachol that produces 50% of the maximal contraction, indicating tissue sensitivity to the agonist. Contractile responses of smooth muscle are expressed as mN/mg of tissue. Data are shown as the mean ± SEM from nine independent experiments. Statistical analyses were performed using unpaired Student’s *t*-tests. * *p* < 0.05 vs. respective control group. V, vehicle.

Detrusor muscle contraction was evaluated using concentration–response curves for carbachol (CCh; 10 nM–100 µM). Pre-incubation with heme (100 µM) significantly reduced (*p* < 0.05) the maximum contractile response (Emax) induced by carbachol compared to control tissues pre-incubated with vehicle (Figure 2A,B), without altering potency (pEC50) values (Figure 2C). This reduction in contractile amplitude reflects a functional relaxation of detrusor smooth muscle.

When tissues were pre-treated with ODQ (10 µM), the inhibitory effect of heme on CCh-induced contraction was abolished, with no significant differences in either Emax or pEC50 compared to control tissues (Figure 2D–F). Similarly, pre-treatment with the HO inhibitor 1J (100 µM) prevented the heme-induced reduction in contractile amplitude and potency (Figure 2G–I). Collectively, these findings demonstrate that heme decreases detrusor responsiveness to muscarinic stimulation through activation of the HO–sGC pathway.

### 3.3. Heme Reduces KCl-Induced Detrusor Contraction via the HO–sGC Pathway

**Figure 3:**
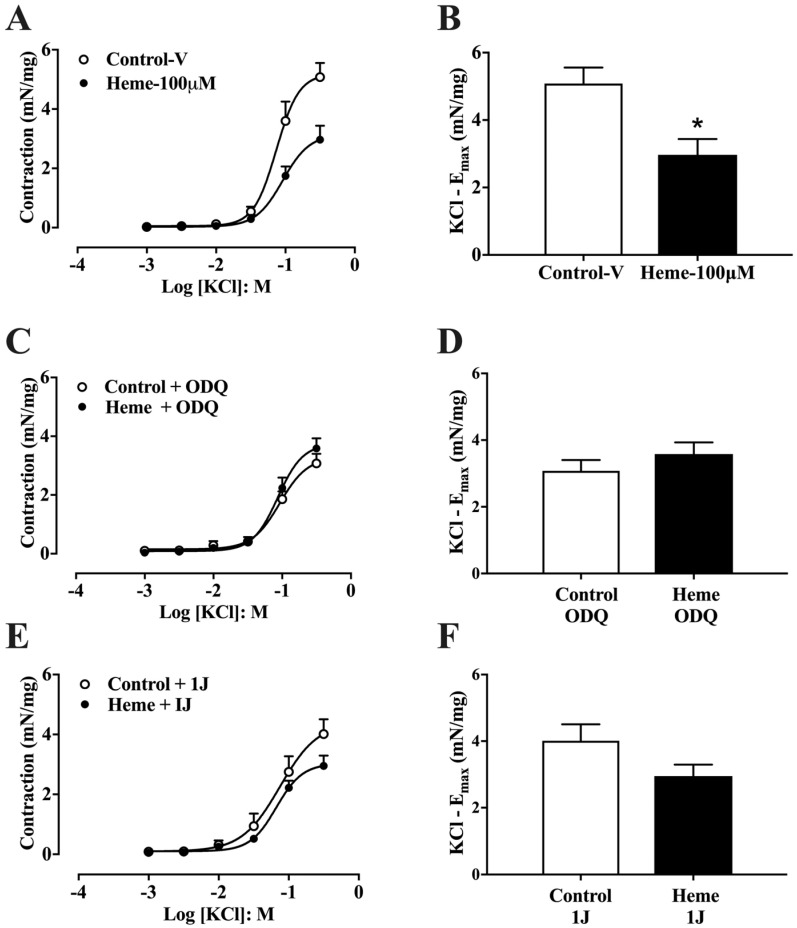
Concentration–response curves to KCl in mouse detrusor smooth muscle: (**A**) Curves obtained in tissues pre-incubated with vehicle (Control–v) or heme (100 µM). (**C**) Curves obtained in the presence of the sGC inhibitor ODQ (10 µM) with or without heme (100 µM). (**E**) Curves obtained in the presence of the HO inhibitor 1J (100 µM) with or without heme (100 µM). (**B**,**D**,**F**) Maximal contractile responses (E_max_). Contractile responses of smooth muscle are expressed as mN/mg of tissue. Data are shown as the mean ± SEM from nine independent experiments. Statistical analyses were performed using unpaired Student’s *t*-tests. * *p* < 0.05 vs. respective control group. V, vehicle.

To assess the effect of heme on non-receptor-mediated contraction, cumulative concentration–response curves to KCl (1–300 mM) were generated. Pre-incubation with heme (100 µM) significantly (*p* < 0.05) reduced the maximal contractile response (Emax) compared with vehicle-treated controls (Figure 3A,B).

However, pre-treatment with ODQ (10 µM; Figure 3C,D) or the HO inhibitor 1J (100 µM; Figure 3E,F) abolished the inhibitory effect of heme on KCl-induced contraction. These results indicate that both heme metabolism via HO and activation of sGC are necessary for the heme-mediated reduction in detrusor contractility, consistent with its relaxant effect.

### 3.4. Heme Reduces Neurogenic Detrusor Contraction via the HO–sGC Pathway

**Figure 4:**
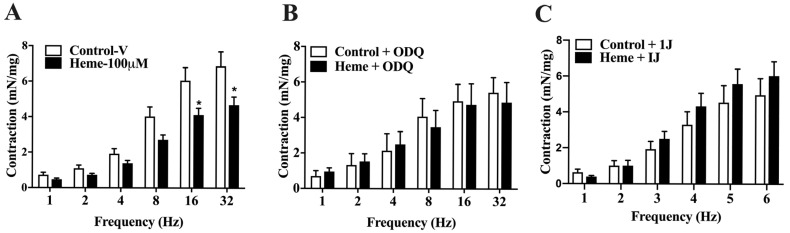
Frequency-dependent contractions of mouse detrusor smooth muscle induced by EFS: (**A**) Contraction responses in tissues pre-incubated with vehicle (Control–v) or heme (100 µM). (**B**) Responses obtained in the presence of the sGC inhibitor ODQ (10 µM) with or without heme (100 µM). (**C**) Responses obtained in the presence of the HO inhibitor 1J (100 µM) with or without heme (100 µM). Contractile responses of smooth muscle are expressed as mN/mg of tissue. Statistical analyses were performed using unpaired Student’s *t*-tests. Data are shown as the mean ± SEM from eight independent experiments. * *p* < 0.05 vs. respective control group. V, vehicle.

Detrusor muscle contraction induced by EFS (1–32 Hz) showed a frequency-dependent response. Pre-incubation with heme (100 µM) significantly reduced (*p* < 0.05) neurogenic contraction responses at higher frequencies (16 and 32 Hz) compared to the control group (Figure 4A).

When detrusor tissues were pre-treated with ODQ (10 µM; Figure 4B) or the HO inhibitor 1J (100 µM; Figure 4C), the inhibitory effect of heme on neurogenic contractions was abolished, confirming the critical role of the HO–CO–sGC–cGMP pathway in mediating heme-induced detrusor relaxation under neurogenic stimulation.

### 3.5. Heme Elevates cGMP Levels in Detrusor Tissue

**Figure 5:**
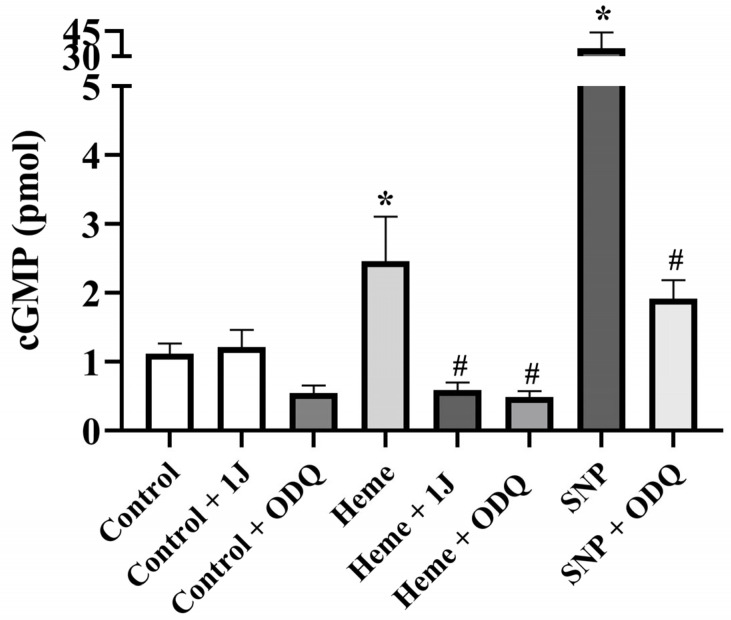
cGMP levels in detrusor tissues from mice. Detrusor strips were incubated with heme (100 µM) in the absence and in the presence of the sGC inhibitor ODQ (100 µM), or the HO inhibitor 1J (100 µM). SNP (100 µM) was used as a positive control in the absence and presence of ODQ (100 µM). cGMP levels represent the mean ± SEM for 4–6 animals in each group. * *p* < 0.05 compared with the control group; # *p* < 0.05 compared with the respective group in the absence of ODQ or 1J.

Basal cGMP concentrations were significantly increased (*p* < 0.05) in detrusor tissues incubated with heme (100 µM) compared with vehicle-treated controls (Figure 5). This effect was markedly attenuated by pre-incubation with either ODQ (100 µM) or 1J (100 µM), confirming the dependence of cGMP elevation on both HO activity and sGC activation.

As a positive control, SNP (100 µM) significantly increased cGMP levels (*p* < 0.05 vs. control), and this effect was completely abolished by ODQ, validating the assay reliability (Figure 5).

## 4. Discussion

Heme is a critical molecule for oxygen transport and cellular respiration; however, it can be a potent pro-oxidant when released in excess during intravascular hemolysis, as observed in SCD. Its accumulation has been implicated in several pathophysiological processes, including inflammation, endothelial dysfunction, and vaso-occlusion [12,39,40]. Despite the well-established roles of heme in vascular and inflammatory mechanisms, its potential contribution to lower urinary tract dysfunction in SCD remains unexplored.

In the present study, we showed that heme directly influences detrusor smooth muscle function ex vivo. The concentration employed (100 µM) was selected to reflect clinically relevant levels reported during episodes of intravascular hemolysis in SCD. A previous study demonstrated that plasma heme concentrations in patients with this condition average 54 ± 25 µM during the steady state and can reach up to approximately 150 µM under more intense hemolytic conditions, supporting the physiological relevance of the concentration used here [41]. These values fall within the pathophysiological range observed in sickle cell disease, strengthening the translational significance of the findings and justifying the experimental design.

Heme metabolism through the HO system generates CO, which acts as a gaseous messenger capable of activating sGC and increasing intracellular cGMP, thereby promoting smooth muscle relaxation [27,31,42]. Previous studies have shown that the addition of exogenous nitric oxide (NO) and/or CO donors induces cGMP-dependent relaxation in corpus cavernosum and urethral smooth muscle from rabbits through sGC activation, thereby modulating smooth muscle tone in these tissues [43]. Likewise, CO infusion has been reported to induce relaxation in human thoracic artery rings via a cGMP-dependent mechanism [44]. More recently, our group demonstrated that heme can induce in vitro relaxation of cavernous smooth muscle in mice through a similar cGMP-mediated mechanism [31,37]. Our findings extend these observations, demonstrating that this mechanism also operates in detrusor tissue. To assess the role of heme and its metabolite CO in detrusor smooth muscle relaxation, we employed pharmacological inhibitors targeting key enzymes in this pathway. The non-selective HO inhibitor 1J and the specific sGC inhibitor ODQ were used as experimental tools to dissect the HO–CO–sGC–cGMP signaling axis in bladder function [45]. Both inhibitors completely abolished heme-induced relaxation, indicating that heme exerts its relaxant effect only after enzymatic degradation and through subsequent CO-mediated activation of sGC. Collectively, these findings support the conclusion that CO generated from heme metabolism acts as a modulator of detrusor smooth muscle tone. This mechanism parallels previously characterized pathways in vascular and erectile tissues and suggests that the HO–CO–sGC–cGMP axis also plays a role in the regulation of bladder contractility.

Bladder function is regulated by a coordinated interaction between sympathetic and parasympathetic pathways, which control urine storage and voiding through complex neural and muscular mechanisms [46,47]. During micturition, detrusor smooth muscle contraction is primarily mediated by acetylcholine released from parasympathetic nerves, acting on muscarinic M3 receptors to promote calcium-dependent contraction [48]. In the present study, we demonstrated that pre-incubation with heme in vitro significantly reduced detrusor contractile responses induced by carbachol, EFS, and KCl. Because KCl evokes contraction through membrane depolarization and calcium influx, independently of receptor activation [49], the consistent inhibitory effect of heme across all stimuli suggests a modulation of intracellular signaling pathways controlling smooth muscle tone. Inhibition of HO with 1J or sGC with ODQ prevented this effect, confirming that heme decreases detrusor contractility through activation of the HO–CO–sGC–cGMP pathway, reproducing a functional pattern similar to that observed in SCD, where excessive intravascular hemolysis leads to elevated circulating heme and bladder hypocontractility [32,50].

Consistent with the functional findings, biochemical analyses showed that cGMP levels were significantly higher in detrusor tissues pre-incubated with heme, while inhibition of HO or sGC prevented this increase. These results confirm that the relaxant effect of heme depends on its metabolism and subsequent activation of the HO–CO–sGC–cGMP pathway. This interpretation is supported by previous studies demonstrating elevated cGMP levels after in vitro exposure to heme in mouse cavernous smooth muscle and following a single intraperitoneal injection of heme in rat penile tissue through an HO-dependent mechanism [31,51]. Together, these findings reinforce the idea that the heme–HO–CO–cGMP axis operates as a conserved regulatory pathway controlling smooth muscle relaxation across different organ systems, including the urinary bladder.

Clinical evidence indicates that CO exposure is associated with voiding dysfunctions, including symptoms such as urinary urgency and incontinence [52,53,54]. These observations are consistent with our in vitro findings and support the notion that CO plays a physiological role in modulating lower urinary tract function. Together, experimental and clinical data suggest that excessive CO generation, either due to exogenous exposure or increased endogenous production from heme metabolism, can alter detrusor activity and contribute to disturbances in bladder control.

In our experimental protocol, detrusor strips were exposed to heme acutely (30 min) to investigate the functional consequences of heme overload and to pharmacologically dissect the downstream signaling pathway, rather than to evaluate transcriptional regulation. Thus, a rapid upregulation of HO-1 is unlikely to have contributed to the observed effects. The complete abolition of heme-induced relaxation and contractile impairment by the HO inhibitor 1J and the sGC inhibitor ODQ indicates that the constitutive HO activity present in the detrusor was sufficient to metabolize exogenous heme and generate CO capable of activating sGC under these conditions. Immunohistochemical studies in the pig bladder have demonstrated that both HO-1 and HO-2 isoforms are expressed in the detrusor, with HO-2 mainly localized in nerve trunks and vascular endothelium and HO-1 in nerve fibers and smooth muscle cells, supporting the presence of a constitutive HO–CO system capable of modulating bladder tone [28]. Although our study did not distinguish between isoforms, the short exposure period favors HO-2 as the predominant contributor. HO-1 typically requires longer durations and stress conditions for induction. It is plausible that HO-2 played a central role in heme degradation and CO generation under our experimental conditions. Furthermore, the heme concentrations used in this study reflect the pathophysiological range observed during hemolytic crises in SCD. Within this range, we observed no loss of tissue reactivity, since inhibition of HO or sGC fully restored contractile responses, suggesting that the detrusor remained viable. Future investigations quantifying HO isoforms and assessing cell viability after prolonged heme exposure will be essential to define the concentration threshold at which heme becomes cytotoxic to detrusor smooth muscle.

Although our study provides strong mechanistic evidence, certain methodological considerations should be acknowledged. First, heme can form π–π and μ–oxo dimers or higher-order aggregates in aqueous environments, depending on concentration and physicochemical conditions, which may reduce the pool of monomeric heme available for enzymatic degradation by HO and slightly influence nonenzymatic interactions with smooth muscle membranes or ion channels [55]. Second, CO production was not directly quantified after heme exposure; thus, although pharmacological inhibition data strongly implicate the HO–CO–sGC–cGMP pathway, direct measurements are needed to confirm CO generation under these experimental conditions. Third, heme degradation activity and free heme concentrations in detrusor tissue were not directly assessed, and future studies quantifying CO or biliverdin formation, HO isoform activity, and residual free heme levels will help define degradation kinetics and clarify the threshold at which heme becomes cytotoxic. Fourth, NO synthase inhibition with L-NAME was not tested, preventing the exclusion of a minor contribution from endogenous NO to the observed effects. Finally, these findings were obtained from in vitro experiments using detrusor tissue from normal mice; in vivo studies employing transgenic sickle cell models will be important to validate these mechanisms under hemolytic and inflammatory conditions.

## 5. Conclusions

**Figure 6:**
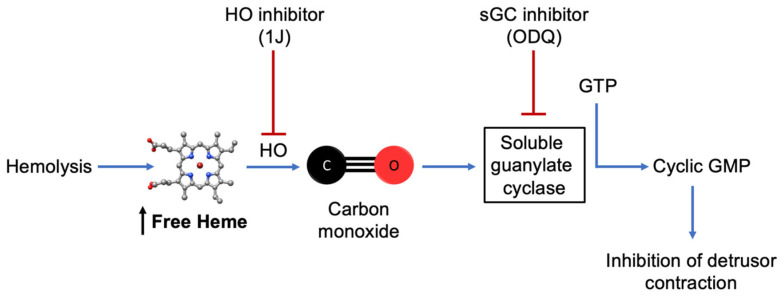
Proposed mechanism of heme-induced detrusor relaxation. Excess free heme, such as that released during intravascular hemolysis, can be metabolized by HO to generate CO. CO activates sGC, increasing cGMP levels and leading to inhibition of detrusor smooth muscle contraction. In this study, ex vivo exposure to heme reproduced this pathway, while pharmacological inhibition of HO (1J) or sGC (ODQ) abolished the effect, confirming the involvement of the HO–CO–sGC–cGMP signaling cascade in bladder relaxation.

This study provides evidence that free heme directly modulates bladder contractility by promoting detrusor smooth muscle relaxation via the HO–CO–sGC–cGMP signaling pathway (Figure 6). In vitro exposure to heme reduced both receptor-dependent and -independent contractions, an effect abolished by inhibition of HO or sGC, confirming the dependence of this mechanism on heme metabolism and CO generation. The increase in cGMP levels further supports this pathway as a key mediator of heme-induced smooth muscle relaxation. These findings reveal a novel role for heme in regulating bladder function and suggest that excessive circulating heme, as occurs in SCD, may contribute to detrusor hypocontractility and voiding dysfunction. Targeting the HO–CO–sGC–cGMP axis could represent a potential therapeutic approach for bladder complications associated with intravascular hemolysis.

## Data Availability

The original contributions presented in this study are included in the article. Further inquiries can be directed to the corresponding author.
